# Loss of Parietal Memory Network Integrity in Alzheimer’s Disease

**DOI:** 10.3389/fnagi.2019.00067

**Published:** 2019-03-27

**Authors:** Yang Hu, Wenying Du, Yiwen Zhang, Ningning Li, Ying Han, Zhi Yang

**Affiliations:** ^1^Shanghai Key Laboratory of Psychotic Disorders, Shanghai Mental Health Center, School of Medicine, Shanghai Jiao Tong University, Shanghai, China; ^2^Brain Science and Technology Research Center, Shanghai Jiao Tong University, Shanghai, China; ^3^Department of Neurology, XuanWu Hospital of Capital Medical University, Beijing, China; ^4^Center of Alzheimer’s Disease, Beijing Institute for Brain Disorders, Beijing, China; ^5^Beijing Institute of Geriatrics, Beijing, China; ^6^National Clinical Research Center for Geriatric Disorders, Beijing, China; ^7^Institute of Psychological and Behavioral Science, Shanghai Jiao Tong University, Shanghai, China

**Keywords:** Alzheimer’s disease, parietal memory network, default mode network, network integrity, independent component analysis

## Abstract

A functional brain network, termed the parietal memory network (PMN), has been shown to reflect the familiarity of stimuli in both memory encoding and retrieval. The function of this network has been separated from the commonly investigated default mode network (DMN) in both resting-state fMRI and task-activations. This study examined the deficit of the PMN in Alzheimer’s disease (AD) patients using resting-state fMRI and independent component analysis (ICA) and investigated its diagnostic value in identifying AD patients. The DMN was also examined as a reference network. In addition, the robustness of the findings was examined using different types of analysis methods and parameters. Our results showed that the integrity as an intrinsic connectivity network for the PMN was significantly decreased in AD and this feature showed at least equivalent predictive ability to that for the DMN. These findings were robust to varied methods and parameters. Our findings suggest that the intrinsic connectivity of the PMN is disrupted in AD and further call for considering the PMN and the DMN separately in clinical neuroimaging studies.

## Introduction

The default mode network (DMN) is the most frequently investigated functional brain network in Alzheimer’s disease (AD), and the intrinsic connectivity of DMN has been reported disrupted in a variety of resting-state fMRI studies (Greicius et al., 2004; Jones et al., 2011, 2016; Petrella et al., 2011). The DMN connectivity disruption has also been identified in populations at risks for AD, such as elderly people, mild cognitive impairment (MCI) patients, and APOE-ε4 allele carriers (Sorg et al., 2007; Damoiseaux et al., 2008; Filippini et al., 2009; Petrella et al., 2011; Binnewijzend et al., 2012). While an increasing number of studies have pointed to the DMN for major functional network deficit in AD, the relationship between the functions of DMN and the early symptoms of AD is still unclear. Clinically AD first targets recent memory function, impairing the ability to remember recently acquired information, while DMN has been named after its characteristics of showing deactivation in attention-demanding tasks, and its functions have been demonstrated in vast cognitive domains (Andrews-Hanna et al., 2010; Raichle, 2015). The posterior part of DMN (i.e., posterior cingulate/precuneus and bilateral parietal cortex) are often linked to memory processing (Andrews-Hanna et al., 2010; Sestieri et al., 2011; Raichle, 2015), but the parietal regions are functionally inhomogeneous and memory functions are not unique to the DMN regions (Cavanna and Trimble, 2006; Cauda et al., 2010; Sestieri et al., 2017).

Recently, a memory-related brain network has been separated from the posterior part of the DMN, termed as the parietal memory network (PMN, Gilmore et al., 2015). Studies have demonstrated that the PMN’s activity is a reflection of the familiarity of a stimulus in both memory encoding and retrieval, and generally across task conditions (Nelson et al., 2013; Gilmore et al., 2015; McDermott et al., 2017). The PMN has been disassociated from the DMN in different types of memory retrieval tasks (McDermott et al., 2009; Chen et al., 2017), where successful recognition memory activates the PMN, while autobiographical retrieval activates the DMN. The PMN includes the middle cingulate cortex (MCC), the precuneus (PCU), and the inferior parietal lobule/angular gyrus (IPL/AG). While these regions are all adjacent to the DMN or even has been attributed to the DMN in some studies (Littow et al., 2010; Damoiseaux et al., 2012; Manoliu et al., 2014; La et al., 2015), the PMN could be reliably separated from the DMN via different methods in resting-state fMRI studies (Yeo et al., 2011; Hu et al., 2016). Further, our previous study has demonstrated that the PMN in resting-state has an obvious age-dependent inter-individual variability, while the DMN does not exhibit age-dependence (Yang et al., 2012, 2014).

The emergence of the PMN has raised our curiosity to consider its role in AD. The double dissociation between the PMN and the DMN in their roles in memory processing leads to our hypothesis that the PMN is also impaired in AD. Further, the spatial adjacency between the PMN and the posterior DMN regions implies a possibility that the deficits previously we attributed to the DMN may also be related to the PMN. The robust separation of the PMN from the DMN in resting-state fMRI provides a chance to examine this hypothesis.

We examined the above hypothesis by comparing the integrity of the PMN between AD patients and healthy controls (HCs) using independent component analysis (ICA). Two commonly used group ICA algorithms were applied and the effects of different model orders (MOs), which is an important yet undetermined parameter in ICA setting, were explored. The value of the PMN integrity in classifying AD patients was further examined. All the analysis were also performed for the DMN, which worked as a reference network.

## Materials and Methods

### Participants

Seventy-nine subjects (36 AD patients and 43 HCs) who were all Han nationality and right-handed participated in this study. AD patients were recruited from a memory clinic of Xuanwu Hospital (Beijing, China). HCs were recruited from a community via advertisements. AD patients met the National Institute of Neurological and Communicative Disorders and Stroke and the AD and Related Disorders Association (NINCDS-ADRDA) criteria for probable AD (McKhann et al., 1984) and the diagnosis of AD was confirmed by more than two professional neurologists. The inclusion criteria for HCs were no memory complaints, no positive sign in the neurological exam. Subjects would be excluded if they met the following conditions: the history of stroke, drug or alcohol abuse, psychiatric disorder or cognitive impairment caused by traumatic brain injury, central nervous system diseases such as brain tumors, Parkinson’s disease, encephalitis or epilepsy, and systemic diseases such as thyroid disease, pernicious anemia, luetic brain disease or Acquired Immune Deficiency Syndrome. All subjects underwent the neuropsychological tests battery including clinical dementia rating (CDR, Morris, 1993), Mini Mental State Exam (MMSE, Folstein et al., 1975), Montreal Cognitive Assessment (MoCA, Nasreddine et al., 2005), Auditory Verbal Learning Task (AVLT, Schmidt, 1996) and activities of daily living (ADL) scale. This study was carried out in accordance with the recommendations of Medical Research Ethics Committee of Xuanwu Hospital, Beijing, China with written informed consent from all subjects. All subjects gave written informed consent in accordance with the Declaration of Helsinki. The protocol was approved by the Medical Research Ethics Committee of Xuanwu Hospital, Beijing, China.

### Data Acquisition

Imaging was performed on an 8-channel head coil, 3.0T Siemens Trio system. T1-weighted structural images were collected with magnetization prepared rapid acquisition (MPRAGE) gradient echo sequence (echo time = 2.2 ms, repetition time = 1,900 ms, inversion time = 900 ms, flip angle = 9°, field of view = 256 mm, matrix = 512 * 512, voxel size = 0.5 * 0.5 * 1 mm^3^ and 176 slices). Resting state images were acquired with a gradient echo planar imaging sequence (repetition time = 2,000 ms, echo time = 40 ms, flip angle = 90°, field of view = 256 mm, matrix = 64 * 64, voxel size = 4 * 4 * 5 mm^3^, 28 slices, and 239 volumes). Participants were informed to lie still with their eyes closed and remain awake.

### Data Preprocessing

Structural images were first processed using Volbrain (Manjón and Coupé, 2016) to extract the brain and segment the brain into gray matter (GM), white matter (WM), cerebrospinal fluid (CSF), and several subcortical structures including the hippocampus. To improve image registration quality, 20 structural brain images (10 images from each group) were randomly selected to build a sample-specific structural template using ANTs (Version 2.1.0, Tustison et al., 2014) and the following group analyses were performed in this customized reference space.

Resting-state fMRI images were analyzed with FSL (Version 5.0.10, Jenkinson et al., 2012). The main procedures consisted of: (1) removing the first five volumes; (2) head motion correction; (3) slice timing correction; (4) grand mean scaling to 10,000; and (5) high pass temporal filtering at 0.01 Hz. The fMRI images were transformed into the customized reference space by a two-step registration scheme using FSL and ANTs: the mid-time point fMRI volume was registered to native space structural image with boundary-based registration (BBR, Greve and Fischl, 2009); then native space structural image was registered to reference space with ANTs’ non-linear transformation. The format conversion between FSL and ANTs were carried out using Convert3D (Yushkevich et al., 2006). These transformed fMRI images were further resampled into 3 * 3 * 3 mm^3^ resolution. We did not perform nuisance regression (Bright and Murphy, 2015), because ICA could split brain networks and artifacts/noises into different components, and thus reduce the influence of non-neural signals for functional network analyses (Pruim et al., 2015a; Du et al., 2016).

Brain extraction, segmentation, and registration accuracy were visually checked. The head motion was lower than 3 mm in translation and 3° in rotation, and the mean frame-wise displacement (meanFD, Power et al., 2012) was lower than 0.5 mm in all subjects.

### Functional Network Analysis

In previous AD-related studies, there were two commonly used group ICA methods: template matching and dual regression (Greicius et al., 2004; Binnewijzend et al., 2012). Both methods need a set of group-level network templates, but they differ in their ways of obtaining individual-level network spatial maps. Template matching would perform ICA for each subject and match individual-level ICs across subjects with the group-level templates (Greicius et al., 2004; Esposito et al., 2008), while dual regression would use the group-level templates as spatial regressors and a two-step multiple linear regression to get the individual-level spatial maps (Filippini et al., 2009; Nickerson et al., 2017). There were other ways of doing group ICA but they were less frequently used (Lee et al., 2008; Yang et al., 2012; Du and Fan, 2013). We used both the template matching and dual regression approaches in examining the functional networks.

#### Creation of Group-Level PMN/DMN Templates

**Figure 1:**
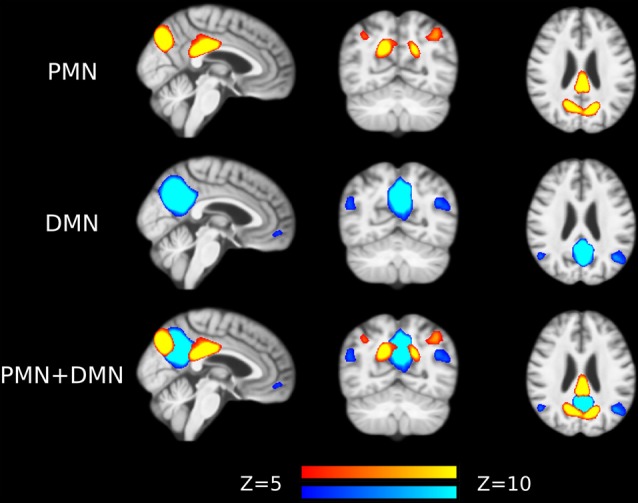
Group-level independent components (ICs) representing parietal memory network (PMN) and default mode network (DMN) overlaid above customized structural template. In the third row, PMN is overlaid above DMN to show their spatial overlapping and differences. The network maps are thresholded to reveal their core regions. The brains are displayed in radiological orientation (left is right).

Preprocessed fMRI images of all subjects were pooled to create a sample-specific PMN/DMN templates using temporal concatenation group ICA (TCGICA) implemented in MELODIC (Calhoun et al., 2001; Beckmann and Smith, 2004; Beckmann et al., 2005). Of note, the default MIGP data reduction was disabled (Smith et al., 2014). Specifically, all individual fMRI images were temporally concatenated and decomposed into a set of group-level ICs. These components reflect the common spatial patterns shared by all or part of subjects. Ninety-nine components were automatically estimated using the Laplacian approximation. ICs representing PMN and DMN were selected by matching with previous templates (Yeo et al., 2011; Hu et al., 2016) using spatial correlation accompanied by visual check. The results were presented in Figure 1.

#### Template Matching

Preprocessed fMRI images of each subject were decomposed into individual-level ICs using MELODIC in native space, and these component maps were then transformed into the reference space (Beckmann and Smith, 2004). The MO of the ICA was automatically estimated for each subject using the Laplacian approximation. Then the absolute Pearson’s correlation coefficient between the individual-level ICs and the PMN/DMN templates were computed. The components with the highest correlation to each template were regarded as the PMN/DMN in each subject. Specifically, a components-by-templates correlation matrix was first computed, and the highest correlation was identified to map a component to a template, and the highest correlation was searched in the remaining pairs to match the other template. The correlation was Fisher-Z transformed and termed as network integrity, as this measure indexes the degree to which individual-level network matches to a network template, with the assumption that the template should reflect the ground truth of an integral (healthy and normally functioning) network and a deviation from the network template indicates there is a loss of integrity. The network integrity is a robust indicator of a general deficit of the functional connectivity within the intrinsic network, but this index cannot specify whether the deficit is located in any specific core regions or the functional connectivity linking the regions. This measure has been commonly used in previous studies (Greicius et al., 2004; Esposito et al., 2008; Du and Fan, 2013; Michael et al., 2014).

#### Dual Regression

Dual regression was performed using FSL’s dual_regression script and all group-level IC maps were used as regressors. Specifically, for each subject, the group-level ICs were used as spatial regressors, and their contributions to the subject’s fMRI data were estimated using a linear model. The contributions were depicted using the corresponding time courses. In the same manner, these time courses were further used as temporal regressors and their contributions to the same fMRI dataset were estimated and represented as a set of spatial maps, each corresponding to a group-level IC. After dual regression, the individual-level spatial maps representing PMN/DMN were selected and the absolute Pearson’s correlation coefficients with group-level ICs were computed (and Fisher-Z transformed) as network integrity measures.

#### Statistical Analysis

The network integrity measures for the PMN and DMN were compared using multiple linear regression between AD and HC groups with age, sex, education years, mean FD and GM volume as covariates. To characterize the diagnostic value of network integrity in AD, we conducted a logistic regression with either PMN’s or DMN’s network integrity, age, sex, education years, meanFD, and GM volume as predictors, and evaluated the performance of the model using leave-one-out cross-validation (LOOCV, Kohavi, 1995). A receiver operating characteristic (ROC) curve was calculated and the area under the curve (AUC) was used to quantify the performance (Zweig and Campbell, 1993; Robin et al., 2011). Furthermore, a voxel-wise comparison was made between groups in individual-level PMN/DMN maps using permutation test (5,000 permutations) and threshold-free cluster enhancement (TFCE) multiple comparison correction (Smith and Nichols, 2009; Winkler et al., 2014). The PMN/DMN templates were thresholded by controlling the local false-discovery rate at *p* < 0.05 using mixture modeling (Beckmann et al., 2005; Filippini et al., 2009) and the voxel-wise hypothesis tests were only performed in the above-threshold brain regions. Age, sex, education years, mean FD and GM volume were also included as covariates in the voxel-wise comparison. The voxel-wise analysis could reveal the local difference of a brain network, while network integrity measures the network as a whole. All statistical analyses were carried out in FSL and R (Version 3.4.4, R Core Team, 2018).

#### Effects of Varied Model Orders

To examine the robustness of the findings, we replicated the above analysis by setting the MO at 50, 60, 70, 80 and 90 in both group-level ICA and individual-level ICA. Specifically, at each level of MO, TCGICA was performed and PMN/DMN templates were selected. For template matching, individual-level ICA was also performed at that MO and individual-level PMN/DMN was selected by matching the group-level PMN/DMN templates. Network integrity measures were computed and the corresponding statistical analyses were carried out at each MO.

### Verification Analysis

#### Inclusion of a Control Network

As we expected that both PMN and DMN should exhibit decreased network integrity and voxel-wise functional connectivity in AD, there might be a concern that whether these decreases were specific to PMN/DMN or a widespread phenomenon. To address this concern, we included the medial visual network (MVN) as a control network and all analysis performed on PMN/DMN were repeated for MVN. The MVN is mainly composed of calcarine sulcus, lingual gyrus and cuneus.

#### Effects of Local Gray Matter Loss

In voxel-wise comparisons, we included global GM volume as a covariate. In order to account for the local changes of GM volume, we also repeated the voxel-wise analysis using voxel-wise GM estimation instead of global GM volume as a regressor. FSL’s FAST (Zhang et al., 2001) module was used to obtain the GM partial volume estimation where the value of each voxel represents the relative GM volume. Then the individual-level GM maps were transformed into the standard space and modulated by the Jacobian determinant of warp field (to compensate the local volume changes caused by non-linear registration). The modulated GM maps were used as a voxel-wise regressor for voxel-wise functional connectivity comparisons.

#### Effects of Out-of-Sample PMN/DMN Templates

In the main analysis, we created sample-specific DMN and PMN templates using all subjects, which could best represent the network features of aged people. Here we also repeated the network integrity analysis using out-of-sample and publicly available PMN and DMN templates, to further confirm the robustness of any findings. Two sets of PMN/DMN templates were utilized, one of which is a 70-component ICA decomposition^1^ from Smith et al. (2009), and the other one is from our previous study (Hu et al., 2016), where the PMN and DMN templates were made by using three different group ICA algorithms^2^. For Smith’s templates, MVN was also included and for Hu’s templates, only PMN and DMN were examined.

#### Effects of Nuisance Regression

Although previous studies (Bright and Murphy, 2015; Pruim et al., 2015a) have pointed out that nuisance regression would remove signals of interest (i.e., brain activity) besides noises and reduce temporal degrees of freedom, nuisance regression as a preprocessing step has been commonly used. We further examined that whether including nuisance regression in data preprocessing would change the results. Nuisance regression was performed before temporal filtering. The nuisance regressors include mean time series of WM and ventricle, Friston’s 24-parameter motion model and motion outliers. The WM and ventricle masks were created by combining individual-level segmentation results and tissue priors from Harvard-Oxford Subcortical Atlas in FSL^3^. The volumes with FD higher than 0.5 mm were treated as motion outliers. Network integrity and voxel-wise functional connectivity analyses of PMN/DMN/MVN were performed for re-preprocessed data.

## Results

### Demographics, Brain Volumetric and Neuropsychological Assessments

The comparison results on demographics, brain volumetric, and neuropsychological assessments are summarized in Table 1. There was no difference in age, sex, and years of education between the AD and HC groups. As expected, the AD group showed decreased volumes of GM (*p* = 0.0052), WM (*p* < 0.001) and hippocampus (*p* < 0.001), and increased CSF volume (*p* < 0.001). The intracranial volume did not differ significantly between groups. For the neuropsychological assessments, the AD group showed worse performance on all scales (*p* < 0.001). As for the mean FD, the mean (standard deviation) was 0.23 (0.092) in AD and 0.23 (0.086) in HC, and there was no significant difference between groups (*t* = 0.33, *df* = 72.51, *p* = 0.74).

**Table 1:** Demographics, brain volumetric and neuropsychological assessments.

Variables	AD (*N* = 36)	HC (*N* = 43)	t/χ^2^	*df*^e^	*p*^f^
**Demographics**					
Age in years	67.97 (7.96)^a^	66.86 (5.56)	0.71	60.98	0.48
Sex (male/female)	11/25	16/27	0.15	1	0.70
Education years	9.36 (4.53)	10.46 (4.72)	1.06	75.55	0.29
**Brain volumetric**					
Intracranial volume (cm^3^)	1306.49 (108.22)	1345.36 (133.02)	1.43	76.95	0.16
Gray matter (%)	47.94 (3.04)	49.76 (2.49)	2.89	67.65	0.0052
White matter (%)	29.46 (2.98)	34.37 (3.32)	6.93	76.61	<0.001
CSF (%)	22.61 (4.29)	15.86 (3.61)	7.48	68.71	<0.001
Hippocampi (%)	0.45 (0.083)	0.58 (0.050)	8.43	55.08	<0.001
**Neuropsychological assessments**					
CDR	0.5/1/2 (3/28/5)^b^	0			–
MMSE	16.89 (6.06)	28.23 (2.01)	10.75	41.45	<0.001
MoCA^c^	12.71 (5.40)	26.06 (3.04)	12.65	51.41	<0.001
AVLT-IR^d^	3.74 (3.38)	12.12 (2.61)	14.57	72.92	<0.001
AVLT-DR^d^	0.97 (1.69)	10.19 (2.78)	18.03	70.76	<0.001

*^a^Data presented as mean (standard deviation). ^b^Three subjects had a CDR of 0.5, 28 subjects had a CDR of 1 and five subjects had a CDR of 2. ^c^Missing data of nine subjects for MoCA. ^d^Auditory Verbal Learning Task-IR (AVLT-IR) and AVRT-DR represent immediate recall and delayed recall of AVLT. Missing data of one subject for AVLT. ^e^df stands for degrees of freedom. ^f^For continuous measures, group difference was assessed using two-sample *t*-test; A χ^2^ test was used to compare male/female proportion*.

### Functional Network Integrity Analysis

#### Template Matching

**Figure 2:**
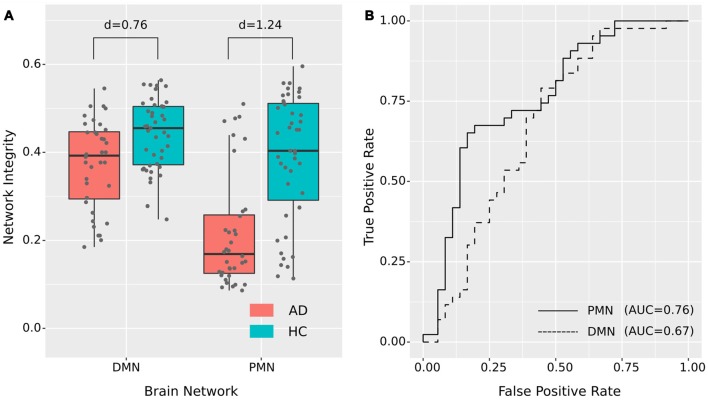
Group difference revealed by network integrity analysis using template matching. **(A)** The difference between Alzheimer’s disease (AD) and healthy control (HC) in PMN’s and DMN’s network integrity. Cohen’s d is shown above each of the boxplots. **(B)** Receiver operating characteristic (ROC) curves for classifying AD from HC using PMN’s or DMN’s network integrity (leave-one-out cross-validated).

As presented in Figure 2 and Table 2, the mean (standard deviation) of the PMN’s network integrity was 0.22 (0.13) for the AD group and 0.39 (0.14) for the HC. Compared to the HC, the AD group exhibited a significant decrease in the PMN’s network integrity (*p* < 0.001) and the effect size (measured as Cohen’s d) was 1.24. Furthermore, the logistic regression model including the PMN’s network integrity had an AUC of 0.76. In comparison, the DMN’s network integrity was 0.37 (0.10) and 0.44 (0.079) for the AD and HC groups respectively. The DMN’s network integrity was also significantly decreased in the AD group (*p* = 0.011), compared with the HC and the effect size is 0.76. The logistic regression model including the DMN’s network integrity had an AUC of 0.67.

**Table 2:** Statistical analysis of network integrity for the PMN and DMN identified using template matching.

Variables	AD	HC	*t*	*p*^c^	Cohen’s d	AUC
**MO = auto^a^**						
PMN	0.22 (0.13)^b^	0.39 (0.14)	4.57	<0.001	1.24	0.76
DMN	0.37 (0.10)	0.44 (0.079)	2.60	<0.011	0.76	0.67
**MO = 50**						
PMN	0.21 (0.11)	0.36 (0.13)	4.75	<0.001	1.26	0.77
DMN	0.42 (0.10)	0.45 (0.12)	0.92	0.36	0.29	0.61
**MO = 60**						
PMN	0.24 (0.13)	0.39 (0.12)	4.14	<0.001	1.15	0.76
DMN	0.39 (0.10)	0.41 (0.13)	0.90	0.37	0.25	0.61
**MO = 70**						
PMN	0.21 (0.12)	0.36 (0.15)	4.06	<0.001	1.12	0.75
DMN	0.42 (0.11)	0.48 (0.087)	2.07	<0.042	0.57	0.63
**MO = 80**						
PMN	0.24 (0.13)	0.40 (0.14)	4.13	<0.001	1.11	0.74
DMN	0.40 (0.10)	0.44 (0.091)	1.64	0.10	0.39	0.63
**MO = 90**						
PMN	0.24 (0.14)	0.39 (0.16)	3.81	<0.001	1.05	0.73
DMN	0.40 (0.11)	0.47 (0.098)	2.37	<0.021	0.64	0.65

*^a^Model order (MO) level was automatically estimated or explicitly specified at 50–90. ^b^Data presented as mean (standard deviation). ^c^The degrees of freedom is 72*.

**Figure 3:**
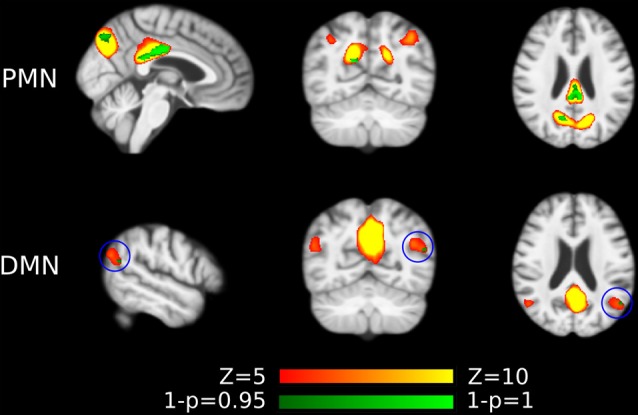
Voxel-wise difference between AD and HC in PMN and DMN spatial maps identified using template matching. Significant clusters (corrected *p* < 0.05) are overlaid above the PMN/DMN spatial maps. The blue circles are used to mark small group difference.

For voxel-wise comparisons, as shown in Figure 3 and Table 3, we measured the ratio of significant voxels in above-threshold regions of each network. There were 401 significant voxels in the PMN, which accounted for 23.03% of the core regions of this network. Correspondingly, there were 11 voxels in the DMN, which accounted for 0.51% in the network.

**Table 3:** Voxel-wise comparison results for the PMN and DMN identified using template matching.

Variables	Total voxel size	Significant voxel size	Ratio of significant voxels (%)	Peak *p* value (corrected)
**MO = auto^a^**				
PMN	1,741^b^	401	23.03	<0.001
DMN	2,144	11	0.51	<0.019
**MO = 50**				
PMN	2,943	252	8.56	<0.001
DMN	5,793	0	0	0.46
**MO = 60**				
PMN	2,812	344	12.23	<0.008
DMN	6,346	0	0	0.48
**MO = 70**				
PMN	2,663	343	12.88	<0.001
DMN	4,264	0	0	<0.064
**MO = 80**				
PMN	2,448	215	8.78	<0.001
DMN	4,179	0	0	0.18
**MO = 90**				
PMN	2,392	483	20.19	<0.001
DMN	2,409	0	0	0.12

*^a^Model order (MO) level was automatically estimated or explicitly specified at 50–90. ^b^Total number of voxels in PMN/DMN networks after thresholded by controlling the local false-discovery rate at *p* < 0.05*.

#### Dual Regression

**Figure 4:**
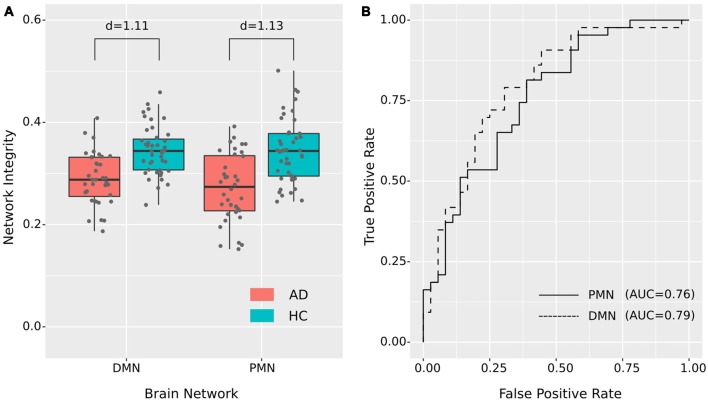
Group difference revealed by network integrity analysis using dual regression. **(A)** The difference between AD and HC in PMN’s and DMN’s network integrity. Cohen’s d is shown above each of the boxplots. **(B)** ROC curves for classifying AD from HC using PMN’s or DMN’s network integrity (leave-one-out cross-validated).

**Figure 5:**
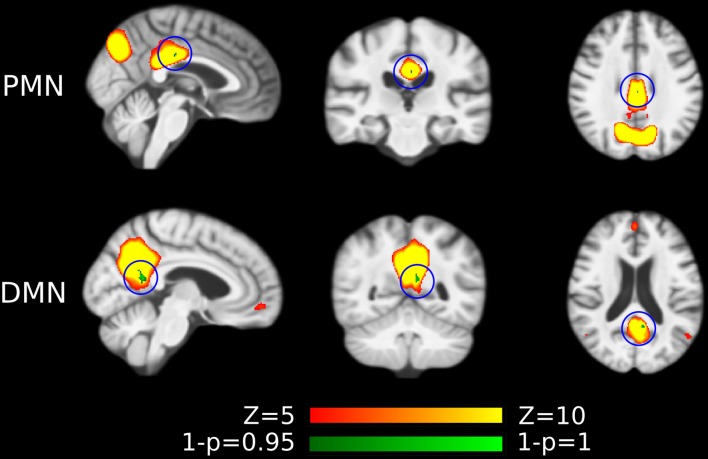
Voxel-wise difference between AD and HC in PMN and DMN spatial maps identified using dual regression. Significant clusters (corrected *p* < 0.05) are overlaid above the PMN/DMN spatial maps. The blue circles are used to mark small group difference.

As presented in Figure 4 and Table 4, the mean (standard deviation) for the PMN’s network integrity was 0.27 (0.067) for AD and 0.34 (0.063) for HC. There was a significant difference between the AD and HC groups (*p* < 0.001) and the effect size was 1.13. In addition, the AUC of PMN in ROC analysis is 0.76. In comparison, the DMN’s network integrity was 0.29 (0.051) for AD and 0.34 (0.048) for HC. The AD-HC group difference was statistically significant (*p* < 0.001) and had an effect size of 1.11. The AUC of DMN is 0.79. For voxel-wise comparisons, shown in Figure 5 and Table 5, the PMN had five significant voxels and a ratio of 0.29%, while the DMN had 17 significant voxels and a ratio of 0.79%.

**Table 4:** Statistical analysis of network integrity for the PMN and DMN identified using dual regression.

Variables	AD	HC	*t*	*p*^c^	Cohen’s d	AUC
**MO = auto^a^**						
PMN	0.27 (0.067)^b^	0.34 (0.063)	4.52	<0.001	1.13	0.76
DMN	0.29 (0.051)	0.34 (0.048)	5.21	<0.001	1.11	0.79
**MO = 50**						
PMN	0.36 (0.087)	0.46 (0.071)	4.86	<0.001	1.23	0.79
DMN	0.42 (0.086)	0.48 (0.076)	3.55	<0.001	0.83	0.72
**MO = 60**						
PMN	0.35 (0.089)	0.44 (0.061)	5.11	<0.001	1.25	0.78
DMN	0.40 (0.082)	0.47 (0.064)	3.99	<0.001	0.89	0.72
**MO = 70**						
PMN	0.33 (0.076)	0.41 (0.064)	4.96	<0.001	1.19	0.78
DMN	0.38 (0.071)	0.43 (0.057)	3.64	<0.001	0.83	0.72
**MO = 80**						
PMN	0.31 (0.076)	0.39 (0.068)	4.70	<0.001	1.13	0.77
DMN	0.37 (0.066)	0.42 (0.066)	3.62	<0.001	0.80	0.70
**MO = 90**						
PMN	0.29 (0.067)	0.37 (0.064)	4.90	<0.001	1.17	0.78
DMN	0.33 (0.059)	0.38 (0.051)	4.80	<0.001	1.03	0.77

*^a^Model order (MO) level was automatically estimated or explicitly specified at 50–90. ^b^Data presented as mean (standard deviation). ^c^The degrees of freedom is 72*.

**Table 5:** Voxel-wise comparison results for the PMN and DMN identified using dual regression.

Variables	Total voxel size	Significant voxel size	Ratio of significant voxels (%)	Peak *p* value (corrected)
**MO = auto^a^**				
PMN	1,741^b^	5	0.29	0.022
DMN	2,144	17	0.79	0.0050
**MO = 50**				
PMN	2,943	4	0.14	0.039
DMN	5,793	41	0.71	<0.001
**MO = 60**				
PMN	2,812	9	0.32	0.010
DMN	6,346	3	0.047	0.034
**MO = 70**				
PMN	2,663	12	0.45	0.0070
DMN	4,264	58	1.36	0.0050
**MO = 80**				
PMN	2,448	19	0.78	0.0020
DMN	4,179	86	2.06	<0.001
**MO = 90**				
PMN	2,392	5	0.21	0.020
DMN	2,409	21	0.87	0.014

*^a^Model order (MO) level was automatically estimated or explicitly specified at 50–90. ^b^Total number of voxels in PMN/DMN networks after thresholded by controlling the local false-discovery rate at p < 0.05*.

#### Effects of Model Order

**Figure 6:**
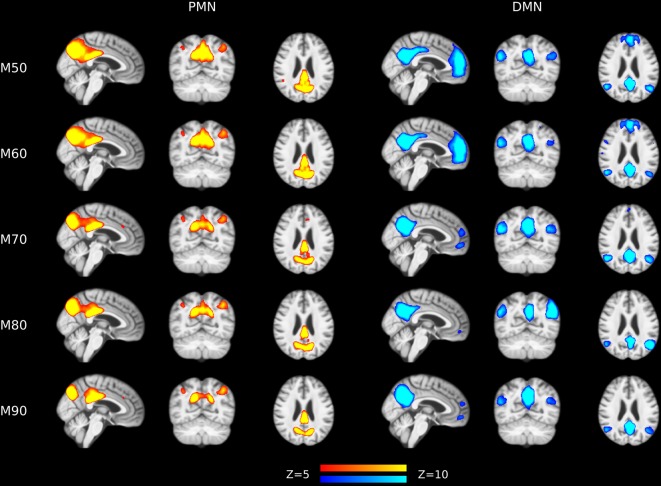
Group-level spatial maps representing PMN and DMN at different model orders overlaid above customized structural template.

The results of different MOs were presented in Tables s 2–5. The group-level PMN/DMN templates were presented in Figure 6. For the network integrity comparison, both template matching and dual regression obtained similar effect sizes and AUC for PMN at different MOs. For the voxel-wise comparison, the results of PMN were similar at varied MOs, while template matching revealed much more significant voxels than dual regression. In comparison, the DMN reflected more variability in both the network integrity and voxel-wise comparisons among MOs in both approaches. For instance, the standard deviation of Cohen’s d at different MOs was 0.20 for the DMN and 0.080 for the PMN in the template matching approach.

### Verification Analysis

#### Inclusion of a Control Network

The group-level spatial maps of MVN were shown in Supplementary Figure S1. The results of network integrity and voxel-wise functional connectivity after including MVN were displayed in Supplementary Tables S1–S4. Different from the PMN/DMN and as expected, the MVN exhibited no significant group difference in both network integrity and voxel-wise functional connectivity.

Therefore, the decreases of PMN/DMN in network integrity and voxel-wise functional connectivity were not widespread phenomena.

#### Effects of Local Gray Matter Loss

**Figure 7:**
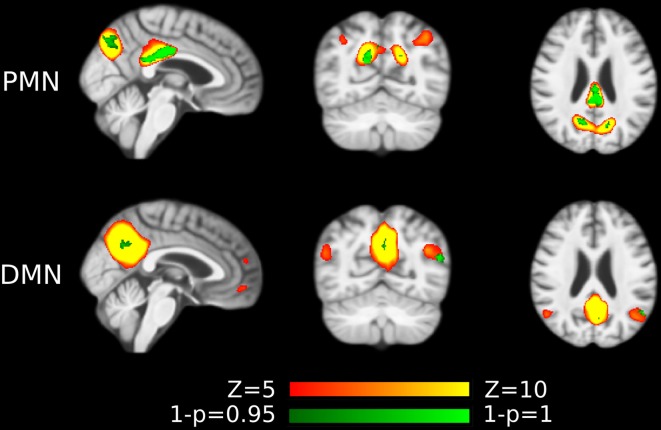
Voxel-wise difference between AD and HC in PMN and DMN spatial maps identified using template matching at automatically estimated model order (MO) after regressing out voxel-wise gray matter (GM) volume. Significant clusters (corrected *p* < 0.05) are overlaid above the PMN/DMN spatial maps.

The voxel-wise comparison results were shown in Figure 7, Supplementary Figure S2 and Supplementary Tables S5, S6. We observed that the inclusion of voxel-wise GM volume would generally increase the size of the significant clusters in both template matching and dual regression approaches. These results together indicated that the functional network abnormality (our findings) was not fully determined by the GM loss.

#### Effects of Out-of-Sample Templates

**Figure 8:**
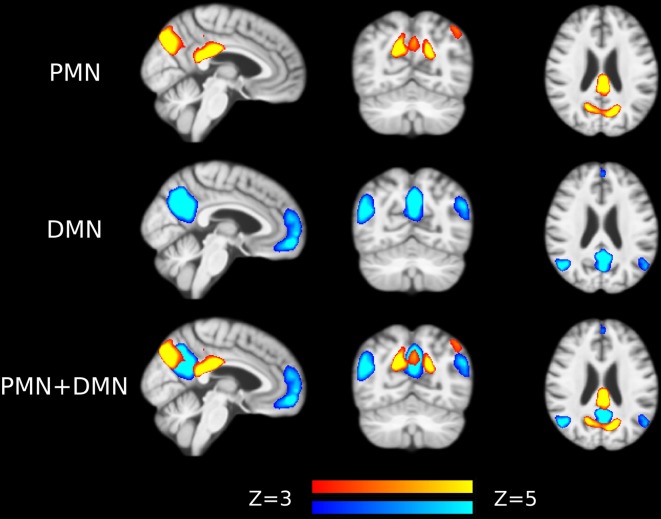
Out-of-sample PMN and DMN templates from Hu et al. (2016).

The spatial maps of Hu’s and Smith’s templates were shown in Figure 8 and Supplementary Figure S3. The network integrity results using out-of-sample templates were presented in Supplementary Tables S7–S10. The group difference we observed before still existed, which further confirmed the robustness of our results. However, we also observed the template-dependent effects. For instance, the individual-level network integrity (correlation) using Smith’s templates was much lower than the sample-specific templates. As neither of the out-of-sample templates was built from aged subjects, these systematic changes probably reflected the different network topological properties in the elderly population.

#### Effects of Nuisance Regression

**Figure 9:**
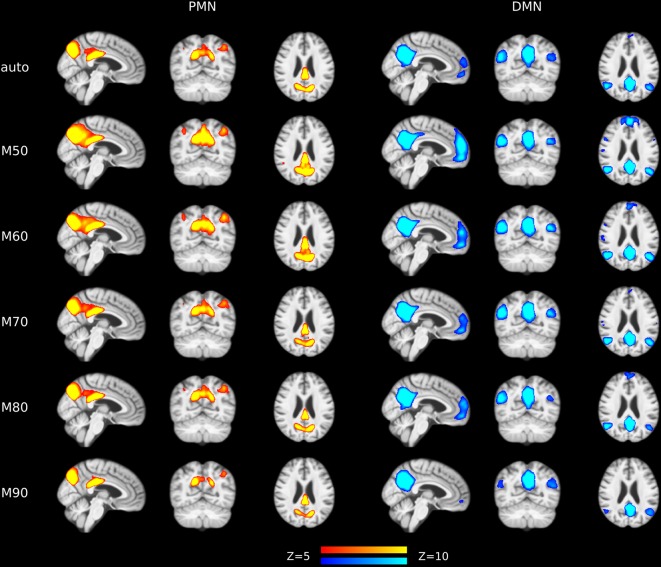
Group-level spatial maps representing PMN and DMN at different model orders after including nuisance regression in data preprocessing.

The group-level spatial maps of PMN/DMN were presented in Figure 9. The results of network integrity and voxel-wise functional connectivity were shown in Supplementary Tables S11–S14. After including the nuisance regression in preprocessing, the group difference identified before could still be identified.

#### Potential Sub-groups of AD

In Figure 2, AD patients appeared to have two clusters in their PMN integrity, with a subset of seven patients exhibiting typical HC integrity. As an exploratory analysis, we compared the seven AD subjects and the remaining 29 patients in three types of variables, including demographic (age/education), volumetric (GM/WM/CSF/hippocampi volume) and neuropsychological assessments (MMSE/MoCA/AVLT). Mean FD was included as a covariate in all comparisons. Detailed statistics on all variables could be found in Supplementary Table S15. The results showed that the 7 AD subjects exhibited significantly higher GM/CSF volumes (*p* = 0.0036/*p* = 0.013) and marginally significantly higher MoCA and MMSE scores (*p* = 0.051/*p* = 0.099) than the other 29 AD subjects. In other words, the 7 AD subjects who had relatively higher PMN integrity also had less GM atrophy and better cognitive performance than the other 29 AD subjects. These results indicated that the integrity of PMN (combined with template matching) is sensitive to the severity of AD symptoms and could be used to detect sub-groups. As the sample sizes of sub-groups were too limited to allow us to establish a reliable association between these variables and the properties of functional networks, these results should be interpreted cautiously and need further validation.

## Discussion

In this study, we found that the PMN had significant lower network integrity in AD, compared with HCs. The PMN had a large effect size and exhibited at least equivalent diagnostic ability to the DMN in identifying AD patients. Further voxel-wise comparisons identified significant decreases in functional connectivity in the core regions of the PMN. These findings were robust at different MOs using two types of group ICA methods. The DMN was analyzed as a reference network, and as expected, exhibited decreased network integrity and voxel-wise functional connectivity in AD group but showed a higher variability across MOs in both methods.

ICA is the most commonly used technique to simultaneously investigate multiple brain networks, which could decompose the fMRI data into a set of inter-independent brain networks en bloc in a data-driven manner (Hyvärinen and Oja, 2000; Beckmann et al., 2005). Due to the inherent order ambiguity of the ICA algorithm, how to apply ICA to multiple subjects for group analysis has been an unsettled issue (Calhoun et al., 2001). Template matching and dual regression are two types of solutions to group ICA and have been commonly used in AD-related studies. Briefly, both methods need a set of brain network templates, but they differ in their ways of obtaining individual-level network spatial maps. Specifically, template matching aligns individual-level ICA results by measuring the similarity to the templates, while dual regression adopts a two-step multiple linear regression to reconstruct individual-level time courses and spatial maps. For the dual regression, the brain network templates are usually created from the sample under study using TCGICA, which would also generate components reflecting artifact/noise, besides brain networks. These artifact/noise components are necessary for dual regression to account for the variances from non-neural sources (Griffanti et al., 2016; Nickerson et al., 2017). Previously, template matching usually only needs the brain network templates of interest (Greicius et al., 2004). For brevity and comparability, here we also use the templates generated from the TCGICA for template matching. These two methods have advantages and disadvantages. For template matching, there are no criteria to determine whether a component is correctly matched to a template. Pearson’s correlation coefficient is a commonly employed similarity measure for template matching, which we adopted in the current study, but there are risks that mismatch could happen, especially when the similarity is low (Zuo et al., 2010). Another concern is the component splitting problem, which means a component could be split into several sub-components, especially in high MO settings (Kiviniemi et al., 2009). Splitting of brain networks is currently thought to reflect the refinement of network functions. In template matching, component splitting could result in incomparability among individual-level ICA results to some degree. In this study, we repeated analysis at different levels of MOs, attempting to mitigate this problem. It is also possible that the component splitting reflects the individuality of brain network activities, which could be reliably detected and vary from person to person. The biggest advantage of template matching is that the individual-level ICA fully preserves the variability of individuals, which are expected to be large, especially in patient populations, because the individual-level ICA maintains the data-driven property and actually we should not make too many assumptions for brain network patterns in real fMRI data. For dual regression, the accuracy of the reconstructed individual-level spatial maps and time courses would be degraded with increased individual variability (Michael et al., 2014; Nickerson et al., 2017). However, dual regression could make group comparison straightforward and showed high test-retest reliability (Zuo et al., 2010). There are theoretically improved group ICA algorithms (Lee et al., 2008; Du and Fan, 2013), though they are scarcely used in AD-related studies. In order to make the results in the current study comparable to those in previous studies, template matching and dual regression were used.

The reliability of fMRI-based studies is a great concern in recent years, therefore we tested many key factors which could potentially influence the results. MO is an undetermined parameter in the ICA algorithm, which could be the most influential factor to any unreliable findings so that a wide range of MOs were tested. Relatively high MOs were selected, based on our previous finding that only in relatively high MO, could PMN and DMN be robustly separated (Hu et al., 2016). The variances explained by PMN could be unintentionally removed in the PCA-based dimension reduction process, when the MO is set at low level (for instance, at 20–30). Nuisance regression is a common technique applied in the preprocessing of resting-state fMRI data, in which WM/ventricle signals and head motion parameters were regressed out before functional network analyses. We did not perform nuisance regression in the main analysis, because ICA could split brain networks and artifacts/noises into different components, and thus reduce the influence of non-neural signals for functional network analyses (Salimi-Khorshidi et al., 2014; Pruim et al., 2015b). ICA-based denoising has been demonstrated to outperform nuisance regression and would be less likely to affect signals of interest (Bright and Murphy, 2015; Pruim et al., 2015a). We also included head motion parameter (i.e., mean FD) as a covariate in statistical analysis to mitigate residual head motion. In verification analysis, we also demonstrated that the findings would not be significantly influenced by nuisance regression. Another notable analysis detail is the avoidance of spatial smoothing during the preprocessing. Spatial smoothing could account for the registration inaccuracy and anatomical variability and thus increase the signal-to-noise ratio. However, common spatial smoothing implementation using isotropic Gaussian kernel filter could potentially blur signals from neighboring brain regions. Due to the anatomical adjacency of PMN and DMN, we did not perform spatial smoothing at the cost of decreased statistical power in the group analysis.

In the current study, the network integrity was measured by the similarity between individual-level networks and group-level network templates. The group-level network templates were made by combining both AD patients and HCs. Making sample-specific group-level templates from all subjects is a common practice for dual regression, which would generate components representing both brain networks of interest and of no interest, as well as artifacts/noises (Griffanti et al., 2016; Nickerson et al., 2017). These components of no interest are important to accurately reconstruct the individual-level spatial maps. Although out-of-sample templates were used in some studies (Jones et al., 2016), there was no systematic examination on the influences of out-of-sample templates, and thus unexpected results could occur. In order to make results from different methods comparable, the group-level network templates used in dual regression were also used for template matching. This kind of group-level templates is not optimal to detect group difference, but we replicated the DMN’s abnormality in our sample, which has been consistently observed in AD patients (Greicius et al., 2004; Binnewijzend et al., 2012), indicating our results are valid (yet conservative). In the verification analysis, we also demonstrated that our results are still valid even if we adopted out-of-sample templates.

Our results call for considering the PMN and the DMN separately in clinical neuroimaging studies. The posterior parietal cortex is not functionally homogenous and plays an important role in episodic memory retrieval (Sestieri et al., 2017). The PMN borders on the DMN in the posterior parietal cortex anatomically, but the PMN is not a sub-component of the (posterior) DMN. These arguments are supported by two lines of previous studies (Gilmore et al., 2015): first, unlike the DMN, the core regions of the PMN are consistently and simultaneously associated with memory encoding and retrieval processes in different task conditions (Kim, 2013; Gilmore et al., 2015). The activity of the PMN is a reflection of the subjective familiarity of a stimulus (McDermott et al., 2017). Specifically, the PMN deactivates in perceiving novel stimuli while activates in familiar ones. This unique pattern differentiates the PMN from other brain networks only involved in certain tasks or in either memory encoding or retrieval. The double disassociation between the PMN and the DMN has been observed in different types of memory tasks (McDermott et al., 2009; Chen et al., 2017). In this regard, the PMN shows a more direct relationship to the early symptoms of AD than the DMN, as DMN is involved in multiple but different cognitive functions. Second, the intrinsic functional connectivity profile of the PMN is distinct from that of the DMN. The PMN and DMN could be separated in resting-state fMRI using graph theoretical methods, clustering, and ICA (Power et al., 2011; Yeo et al., 2011; Yang et al., 2012, 2014; Hu et al., 2016). Unlike the DMN, which could be split into sub-networks in high order model ICA, the PMN was immune from the effects of high MO (Kiviniemi et al., 2009; Hu et al., 2016). These evidence indicated that the PMN is not a subordinate of the DMN but an independent functional brain network. There has been a tendency to attribute any medial and lateral parietal regions to the DMN in some studies, even in situations where network analysis was not performed. Due to the spatial proximity of the PMN and the DMN, it is very likely that some findings located in posterior parietal regions have been attributed to the DMN for interpretation. It is also very possible that posterior parietal cortex contains other functional networks, besides the PMN and the DMN. Amyloid deposition, metabolism reduction, and atrophy are also co-occurring in the posterior parietal cortex at the stage of AD (Buckner et al., 2005). This line of evidence that has been used to support the dysfunction of the DMN could also support the dysfunction of the PMN, and needs further clarification using fine-grained parcellation of the posterior parietal cortex.

As AD is an irreversible process, much endeavor has been made in seeking biomarkers to diagnose AD at an earlier stage. In this study, we examined the ability of the PMN in discriminating AD group from the HC group and found that the PMN had a large effect size and at least equivalent predictability to the DMN. Further study on the prodromal stage of AD and longitudinal analysis are needed to clarify which brain network comes first, although the PMN and DMN were both disrupted in AD. We speculate that due to its close association with memory functions, the dysfunction of the PMN may emerge earlier, with the decline of memory performance before a clinical diagnosis of AD has arrived. In addition, there are different methods for network analysis besides ICA, which often make different assumptions on data and have both advantages and disadvantages. Converging evidence from different types of methods could further confirm the findings of the current study.

In conclusion, the current findings show that the integrity of the PMN is disrupted in AD, and that the PMN have a large effect size and at least equivalent diagnostic ability to the DMN in discriminating AD patients from HCs.

## Author Contributions

ZY, YHu and YHan designed the study. WD collected the data. YHu and NL analyzed the data. All authors contributed to manuscript writing.

## Conflict of Interest Statement

The authors declare that the research was conducted in the absence of any commercial or financial relationships that could be construed as a potential conflict of interest.

## Footnotes

^1^http://www.fmrib.ox.ac.uk/datasets/brainmap+rsns/

^2^https://github.com/yangzhi-psy/PMN-and-DMN-ICA-template

^3^https://fsl.fmrib.ox.ac.uk/fsl/fslwiki/Atlases
