# Supplementary material for: Loss of Parietal Memory Network Integrity in Alzheimer’s Disease
Source: Front Aging Neurosci. 2019 Mar 27;11:67. doi: 10.3389/fnagi.2019.00067 (PMC6446948; doi:10.3389/fnagi.2019.00067)
Supplement: Supplementary file 1 [file Data_Sheet_1.docx]

Supplementary Materials


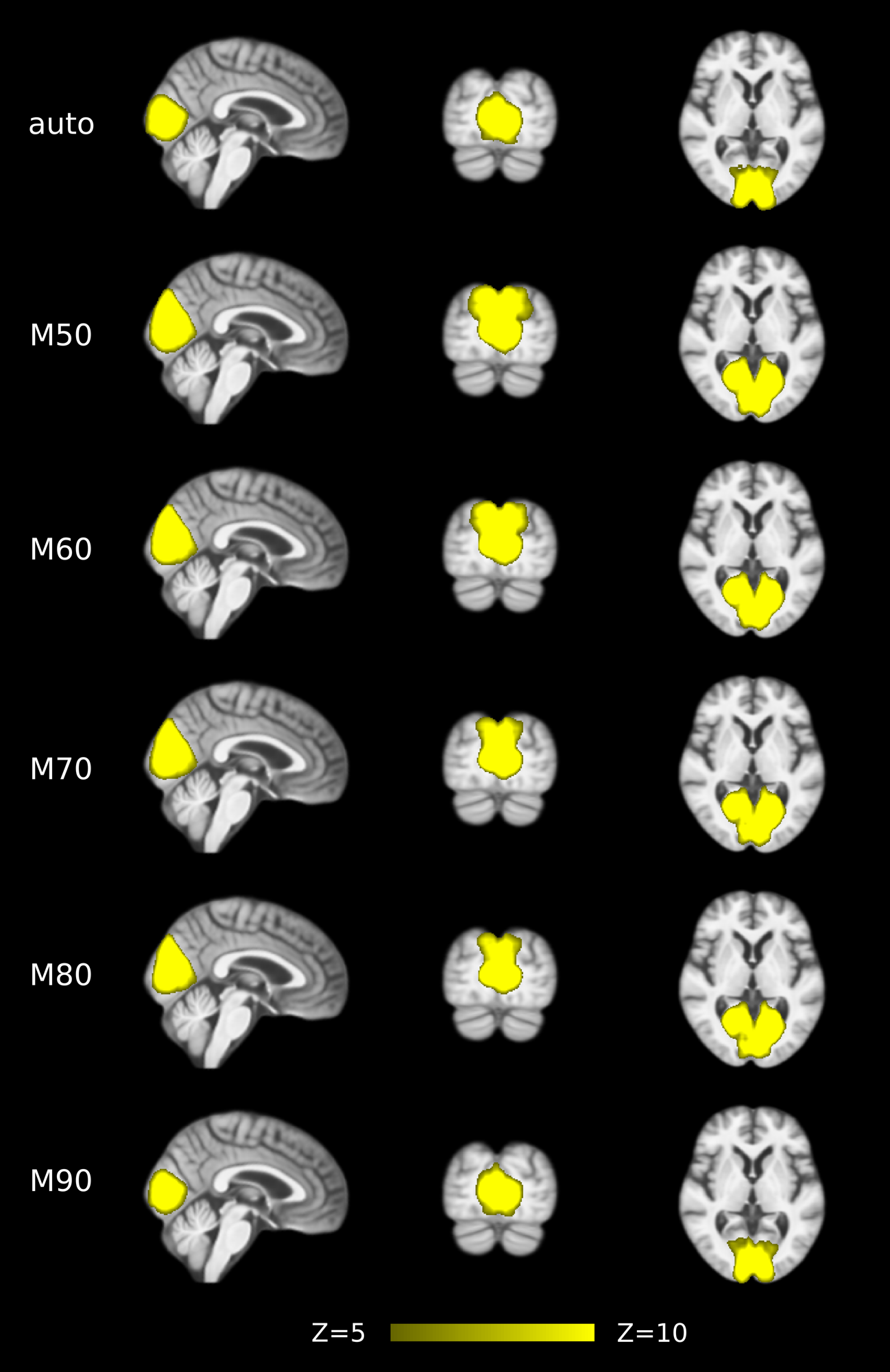


Figure S1. Group-level spatial maps representing medial visual network (MVN) at different model orders (auto-estimated or specified at 50-90).

Table S1. Statistical analysis of network integrity for the PMN/DMN/MVN identified using template matching.

| Variables | AD | HC | t | p^c^ | Cohen’s d | AUC |
| --- | --- | --- | --- | --- | --- | --- |
| **MO = auto^a^** |  |  |  |  |  |  |
| PMN | 0.22 (0.13)^b^ | 0.39 (0.14) | 4.57 | <0.001 | 1.24 | 0.76 |
| DMN | 0.37 (0.10) | 0.44 (0.079) | 2.60 | 0.011 | 0.76 | 0.67 |
| MVN | 0.28 (0.11) | 0.33 (0.16) | 1.21 | 0.23 | 0.38 | 0.62 |
| **MO = 50** |  |  |  |  |  |  |
| PMN | 0.20 (0.11) | 0.36 (0.14) | 4.56 | <0.001 | 1.23 | 0.76 |
| DMN | 0.42 (0.10) | 0.45 (0.12) | 0.92 | 0.36 | 0.29 | 0.61 |
| MVN | 0.39 (0.18) | 0.44 (0.21) | 0.26 | 0.80 | 0.25 | 0.60 |
| **MO = 60** |  |  |  |  |  |  |
| PMN | 0.24 (0.13) | 0.38 (0.13) | 4.09 | <0.001 | 1.14 | 0.76 |
| DMN | 0.39 (0.10) | 0.41 (0.13) | 0.90 | 0.37 | 0.25 | 0.61 |
| MVN | 0.39 (0.18) | 0.46 (0.21) | 0.48 | 0.63 | 0.32 | 0.60 |
| **MO = 70** |  |  |  |  |  |  |
| PMN | 0.21 (0.12) | 0.36 (0.15) | 4.18 | <0.001 | 1.15 | 0.76 |
| DMN | 0.42 (0.11) | 0.48 (0.087) | 2.07 | 0.042 | 0.57 | 0.63 |
| MVN | 0.36 (0.17) | 0.44 (0.20) | 0.87 | 0.39 | 0.41 | 0.60 |
| **MO = 80** |  |  |  |  |  |  |
| PMN | 0.24 (0.14) | 0.40 (0.14) | 4.23 | <0.001 | 1.14 | 0.75 |
| DMN | 0.40 (0.10) | 0.44 (0.091) | 1.64 | 0.10 | 0.39 | 0.63 |
| MVN | 0.36 (0.16) | 0.43 (0.21) | 0.85 | 0.40 | 0.41 | 0.60 |
| **MO = 90** |  |  |  |  |  |  |
| PMN | 0.24 (0.14) | 0.39 (0.16) | 3.81 | <0.001 | 1.05 | 0.73 |
| DMN | 0.40 (0.11) | 0.47 (0.098) | 2.37 | 0.021 | 0.64 | 0.65 |
| MVN | 0.27 (0.11) | 0.33 (0.15) | 1.65 | 0.10 | 0.45 | 0.63 |

^a^ Model order (MO) level was automatically estimated, or explicitly specified at 50-90.

^b^ Data presented as mean (standard deviation).

^c^ The degrees of freedom is 72.

Table S2. Voxel-wise comparison results for the PMN/DMN/MVN identified using template matching.

| Variables | Total Voxel Size | Significant Voxel Size | Ratio of Significant Voxels (%) | Peak p value (corrected) |
| --- | --- | --- | --- | --- |
| **MO = auto^a^** |  |  |  |  |
| PMN | 1741^b^ | 401 | 23.03 | <0.001 |
| DMN | 2144 | 11 | 0.51 | 0.019 |
| MVN | 2651 | 0 | 0 | 0.20 |
| **MO = 50** |  |  |  |  |
| PMN | 2943 | 273 | 9.28 | 0.0018 |
| DMN | 5793 | 0 | 0 | 0.46 |
| MVN | 3524 | 0 | 0 | 0.26 |
| **MO = 60** |  |  |  |  |
| PMN | 2812 | 308 | 10.95 | 0.0010 |
| DMN | 6346 | 0 | 0 | 0.49 |
| MVN | 3208 | 0 | 0 | 0.35 |
| **MO = 70** |  |  |  |  |
| PMN | 2663 | 332 | 12.45 | <0.001 |
| DMN | 4264 | 0 | 0 | 0.064 |
| MVN | 3092 | 0 | 0 | 0.48 |
| **MO = 80** |  |  |  |  |
| PMN | 2448 | 221 | 9.03 | <0.001 |
| DMN | 4179 | 0 | 0 | 0.18 |
| MVN | 3000 | 0 | 0 | 0.32 |
| **MO = 90** |  |  |  |  |
| PMN | 2392 | 442 | 18.48 | <0.001 |
| DMN | 2409 | 0 | 0 | 0.12 |
| MVN | 2429 | 0 | 0 | 0.25 |

^a^ Model order (MO) level was automatically estimated, or explicitly specified at 50-90.

^b^ Total number of voxels in PMN/DMN networks after thresholded by controlling the local false-discovery rate at p < 0.05.

Table S3. Statistical analysis of network integrity for the PMN/DMN/MVN identified using dual regression.

| Variables | AD | HC | t | p^c^ | Cohen’s d | AUC |
| --- | --- | --- | --- | --- | --- | --- |
| **MO = auto^a^** |  |  |  |  |  |  |
| PMN | 0.27 (0.067)^b^ | 0.34 (0.063) | 4.52 | <0.001 | 1.13 | 0.76 |
| DMN | 0.29 (0.051) | 0.34 (0.048) | 5.21 | <0.001 | 1.11 | 0.79 |
| MVN | 0.32 (0.093) | 0.35 (0.091) | 1.03 | 0.31 | 0.28 | 0.60 |
| **MO = 50** |  |  |  |  |  |  |
| PMN | 0.36 (0.087) | 0.46 (0.071) | 4.86 | <0.001 | 1.23 | 0.79 |
| DMN | 0.42 (0.086) | 0.48 (0.076) | 3.55 | <0.001 | 0.83 | 0.72 |
| MVN | 0.42 (0.14) | 0.46 (0.16) | 0.52 | 0.61 | 0.25 | 0.60 |
| **MO = 60** |  |  |  |  |  |  |
| PMN | 0.35 (0.089) | 0.44 (0.061) | 5.11 | <0.001 | 1.25 | 0.79 |
| DMN | 0.40 (0.082) | 0.47 (0.064) | 3.99 | <0.001 | 0.89 | 0.72 |
| MVN | 0.41 (0.13) | 0.45 (0.16) | 0.62 | 0.54 | 0.28 | 0.60 |
| **MO = 70** |  |  |  |  |  |  |
| PMN | 0.33 (0.076) | 0.41 (0.064) | 4.96 | <0.001 | 1.19 | 0.78 |
| DMN | 0.38 (0.071) | 0.43 (0.057) | 3.64 | <0.001 | 0.83 | 0.72 |
| MVN | 0.38 (0.12) | 0.43 (0.14) | 0.91 | 0.37 | 0.33 | 0.60 |
| **MO = 80** |  |  |  |  |  |  |
| PMN | 0.31 (0.076) | 0.39 (0.068) | 4.70 | <0.001 | 1.13 | 0.77 |
| DMN | 0.37 (0.066) | 0.42 (0.066) | 3.62 | <0.001 | 0.80 | 0.70 |
| MVN | 0.37 (0.11) | 0.41 (0.13) | 0.80 | 0.43 | 0.34 | 0.60 |
| **MO = 90** |  |  |  |  |  |  |
| PMN | 0.29 (0.067) | 0.37 (0.064) | 4.90 | <0.001 | 1.17 | 0.78 |
| DMN | 0.33 (0.059) | 0.38 (0.051) | 4.80 | <0.001 | 1.03 | 0.77 |
| MVN | 0.32 (0.090) | 0.34 (0.083) | 1.08 | 0.29 | 0.33 | 0.61 |

^a^ Model order (MO) level was automatically estimated, or explicitly specified at 50-90.

^b^ Data presented as mean (standard deviation).

^c^ The degrees of freedom is 72.

Table S4. Voxel-wise comparison results for the PMN/DMN/MVN identified using dual regression.

| Variables | Total Voxel Size | Significant Voxel Size | Ratio of Significant Voxels (%) | Peak p value (corrected) |
| --- | --- | --- | --- | --- |
| **MO = auto^a^** |  |  |  |  |
| PMN | 1741^b^ | 5 | 0.29 | 0.022 |
| DMN | 2144 | 17 | 0.79 | 0.0052 |
| MVN | 2651 | 0 | 0 | 0.78 |
| **MO = 50** |  |  |  |  |
| PMN | 2943 | 4 | 0.14 | 0.039 |
| DMN | 5793 | 41 | 0.71 | 0.0010 |
| MVN | 3524 | 0 | 0 | 0.55 |
| **MO = 60** |  |  |  |  |
| PMN | 2812 | 9 | 0.32 | 0.010 |
| DMN | 6346 | 3 | 0.047 | 0.034 |
| MVN | 3208 | 0 | 0 | 0.68 |
| **MO = 70** |  |  |  |  |
| PMN | 2663 | 12 | 0.45 | 0.0074 |
| DMN | 4264 | 58 | 1.36 | 0.0046 |
| MVN | 3092 | 0 | 0 | 0.96 |
| **MO = 80** |  |  |  |  |
| PMN | 2448 | 19 | 0.78 | 0.0022 |
| DMN | 4179 | 86 | 2.06 | <0.001 |
| MVN | 3000 | 0 | 0 | 0.84 |
| **MO = 90** |  |  |  |  |
| PMN | 2392 | 5 | 0.21 | 0.020 |
| DMN | 2409 | 21 | 0.87 | 0.014 |
| MVN | 2429 | 0 | 0 | 0.94 |

^a^ Model order (MO) level was automatically estimated, or explicitly specified at 50-90.

^b^ Total number of voxels in PMN/DMN networks after thresholded by controlling the local false-discovery rate at p < 0.05

Table S5. Voxel-wise comparison results for the PMN and DMN identified using template matching after regressing out voxel-wise gray matter volume.

| Variables | Total Voxel Size | Significant Voxel Size | Ratio of Significant Voxels (%) | Peak p value (corrected) |
| --- | --- | --- | --- | --- |
| **MO = auto^a^** |  |  |  |  |
| PMN | 1741^b^ | 494 | 28.37 | <0.001 |
| DMN | 2144 | 85 | 3.96 | 0.0020 |
| **MO = 50** |  |  |  |  |
| PMN | 2943 | 348 | 11.82 | <0.001 |
| DMN | 5793 | 0 | 0 | 0.22 |
| **MO = 60** |  |  |  |  |
| PMN | 2812 | 530 | 18.85 | <0.001 |
| DMN | 6346 | 0 | 0 | 0.21 |
| **MO = 70** |  |  |  |  |
| PMN | 2663 | 526 | 19.75 | <0.001 |
| DMN | 4264 | 32 | 0.75 | 0.0016 |
| **MO = 80** |  |  |  |  |
| PMN | 2448 | 313 | 12.79 | <0.001 |
| DMN | 4179 | 7 | 0.17 | 0.028 |
| **MO = 90** |  |  |  |  |
| PMN | 2392 | 610 | 25.50 | <0.001 |
| DMN | 2409 | 22 | 0.91 | 0.010 |

^a^ Model order (MO) level was automatically estimated, or explicitly specified at 50-90.

^b^ Total number of voxels in PMN/DMN networks after thresholded by controlling the local false-discovery rate at p < 0.05.


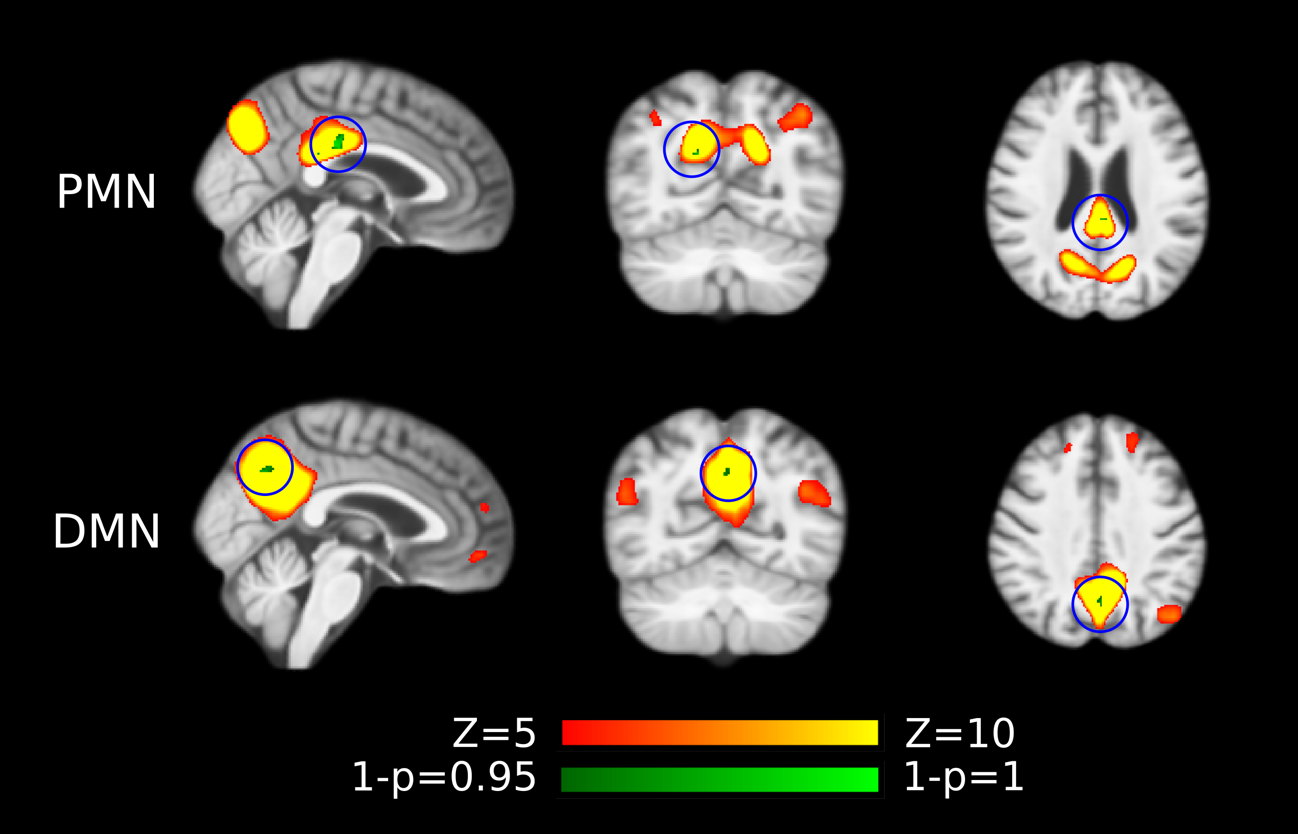


Figure S2. Voxel-wise difference between AD and HC in PMN and DMN spatial maps identified using dual regression at automatically estimated model order after regressing out voxel-wise gray matter volume. Significant clusters (corrected p < 0.05) are overlaid above the PMN/DMN spatial maps. The blue circles are used to mark small group difference.

Table S6. Voxel-wise comparison results for the PMN and DMN identified using dual regression after regressing out voxel-wise gray matter volume.

| Variables | Total Voxel Size | Significant Voxel Size | Ratio of Significant Voxels (%) | Peak p value (corrected) |
| --- | --- | --- | --- | --- |
| **MO = auto^a^** |  |  |  |  |
| PMN | 1741^b^ | 25 | 1.44 | 0.0040 |
| DMN | 2144 | 20 | 0.93 | 0.0072 |
| **MO = 50** |  |  |  |  |
| PMN | 2943 | 11 | 0.37 | 0.0096 |
| DMN | 5793 | 51 | 0.88 | 0.0016 |
| **MO = 60** |  |  |  |  |
| PMN | 2812 | 24 | 0.85 | <0.001 |
| DMN | 6346 | 2 | 0.032 | 0.024 |
| **MO = 70** |  |  |  |  |
| PMN | 2663 | 50 | 1.88 | <0.001 |
| DMN | 4264 | 54 | 1.27 | 0.0036 |
| **MO = 80** |  |  |  |  |
| PMN | 2448 | 49 | 2.00 | <0.001 |
| DMN | 4179 | 96 | 2.30 | <0.001 |
| **MO = 90** |  |  |  |  |
| PMN | 2392 | 19 | 0.79 | 0.0010 |
| DMN | 2409 | 39 | 1.62 | 0.0054 |

^a^ Model order (MO) level was automatically estimated, or explicitly specified at 50-90.

^b^ Total number of voxels in PMN/DMN networks after thresholded by controlling the local false-discovery rate at p < 0.05


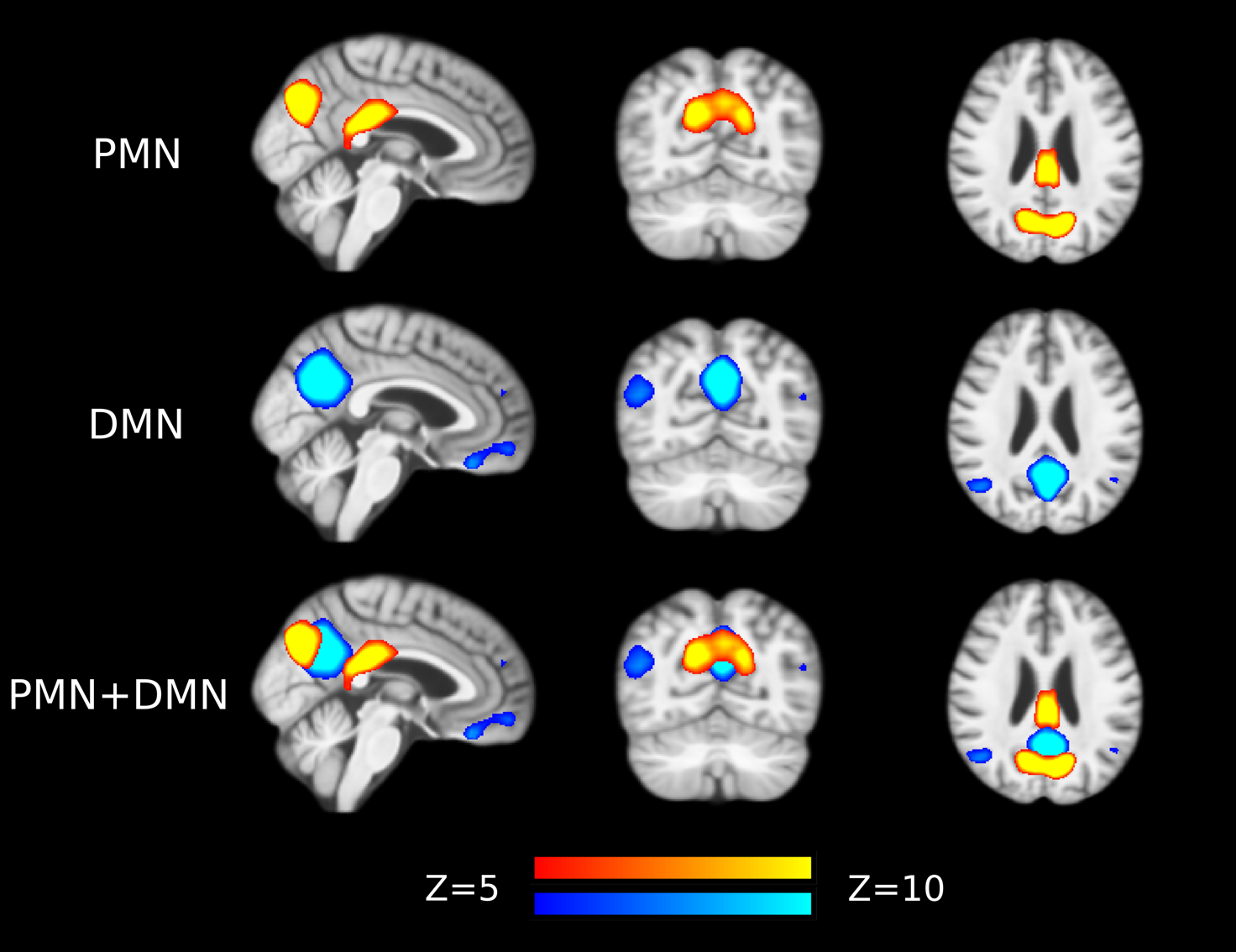


Figure S3. Out-of-sample PMN and DMN templates from Smith et al. (2009).

Table S7. Statistical analysis of network integrity for the PMN/DMN/MVN identified using template matching and Smith’s templates.

| Variables | AD | HC | t | p^c^ | Cohen’s d | AUC |
| --- | --- | --- | --- | --- | --- | --- |
| **MO = auto^a^** |  |  |  |  |  |  |
| PMN | 0.19 (0.11)^b^ | 0.33 (0.11) | 4.63 | <0.001 | 1.24 | 0.75 |
| DMN | 0.33 (0.084) | 0.38 (0.062) | 2.19 | 0.032 | 0.69 | 0.65 |
| MVN | 0.27 (0.10) | 0.32 (0.13) | 1.04 | 0.30 | 0.45 | 0.60 |
| **MO = 50** |  |  |  |  |  |  |
| PMN | 0.12 (0.050) | 0.21 (0.10) | 4.15 | <0.001 | 1.08 | 0.74 |
| DMN | 0.30 (0.094) | 0.36 (0.071) | 3.13 | 0.0026 | 0.73 | 0.68 |
| MVN | 0.23 (0.10) | 0.28 (0.12) | 0.90 | 0.37 | 0.40 | 0.60 |
| **MO = 60** |  |  |  |  |  |  |
| PMN | 0.15 (0.074) | 0.24 (0.11) | 3.29 | 0.0016 | 0.87 | 0.70 |
| DMN | 0.31 (0.092) | 0.37 (0.077) | 2.67 | 0.0095 | 0.65 | 0.67 |
| MVN | 0.25 (0.10) | 0.30 (0.13) | 1.17 | 0.24 | 0.49 | 0.61 |
| **MO = 70** |  |  |  |  |  |  |
| PMN | 0.17 (0.086) | 0.27 (0.12) | 3.38 | 0.0012 | 0.96 | 0.71 |
| DMN | 0.33 (0.091) | 0.38 (0.059) | 2.73 | 0.0079 | 0.70 | 0.67 |
| MVN | 0.25 (0.10) | 0.32 (0.13) | 1.44 | 0.15 | 0.54 | 0.62 |
| **MO = 80** |  |  |  |  |  |  |
| PMN | 0.18 (0.10) | 0.30 (0.13) | 3.62 | <0.001 | 1.00 | 0.72 |
| DMN | 0.33 (0.087) | 0.38 (0.065) | 2.48 | 0.015 | 0.65 | 0.67 |
| MVN | 0.26 (0.10) | 0.32 (0.14) | 1.42 | 0.16 | 0.53 | 0.61 |
| **MO = 90** |  |  |  |  |  |  |
| PMN | 0.20 (0.11) | 0.31 (0.13) | 3.40 | 0.0011 | 0.93 | 0.71 |
| DMN | 0.33 (0.085) | 0.38 (0.065) | 2.79 | 0.0068 | 0.72 | 0.68 |
| MVN | 0.26 (0.10) | 0.34 (0.14) | 1.64 | 0.10 | 0.61 | 0.63 |

^a^ Model order (MO) level was automatically estimated, or explicitly specified at 50-90.

^b^ Data presented as mean (standard deviation).

^c^ The degrees of freedom is 72.

Table S8. Statistical analysis of network integrity for the PMN/DMN/MVN identified using dual regression and Smith’s templates.

| Variables | AD | HC | t | p^c^ | Cohen’s d | AUC |
| --- | --- | --- | --- | --- | --- | --- |
| **MO = auto^a^** |  |  |  |  |  |  |
| PMN | 0.20 (0.051)^b^ | 0.25 (0.046) | 4.50 | <0.001 | 1.13 | 0.75 |
| DMN | 0.19 (0.038) | 0.23 (0.029) | 5.28 | <0.001 | 1.20 | 0.80 |
| MVN | 0.16 (0.050) | 0.17 (0.044) | 1.38 | 0.17 | 0.37 | 0.61 |
| **MO = 50** |  |  |  |  |  |  |
| PMN | 0.22 (0.063) | 0.29 (0.054) | 5.09 | <0.001 | 1.23 | 0.79 |
| DMN | 0.19 (0.039) | 0.23 (0.036) | 4.72 | <0.001 | 0.98 | 0.79 |
| MVN | 0.27 (0.090) | 0.30 (0.11) | 0.76 | 0.45 | 0.31 | 0.60 |
| **MO = 60** |  |  |  |  |  |  |
| PMN | 0.24 (0.069) | 0.31 (0.051) | 4.84 | <0.001 | 1.16 | 0.78 |
| DMN | 0.20 (0.039) | 0.24 (0.030) | 5.44 | <0.001 | 1.17 | 0.79 |
| MVN | 0.27 (0.088) | 0.30 (0.10) | 0.74 | 0.46 | 0.32 | 0.60 |
| **MO = 70** |  |  |  |  |  |  |
| PMN | 0.23 (0.063) | 0.30 (0.056) | 4.47 | <0.001 | 1.08 | 0.76 |
| DMN | 0.24 (0.046) | 0.29 (0.036) | 4.95 | <0.001 | 1.08 | 0.79 |
| MVN | 0.26 (0.088) | 0.29 (0.098) | 0.90 | 0.37 | 0.34 | 0.61 |
| **MO = 80** |  |  |  |  |  |  |
| PMN | 0.23 (0.060) | 0.30 (0.055) | 4.51 | <0.001 | 1.10 | 0.76 |
| DMN | 0.17 (0.031) | 0.20 (0.031) | 4.69 | <0.001 | 1.00 | 0.78 |
| MVN | 0.25 (0.081) | 0.28 (0.093) | 0.76 | 0.45 | 0.34 | 0.60 |
| **MO = 90** |  |  |  |  |  |  |
| PMN | 0.21 (0.054) | 0.27 (0.050) | 4.58 | <0.001 | 1.12 | 0.76 |
| DMN | 0.21 (0.042) | 0.25 (0.029) | 5.66 | <0.001 | 1.21 | 0.81 |
| MVN | 0.17 (0.049) | 0.19 (0.043) | 1.38 | 0.17 | 0.41 | 0.62 |

^a^ Model order (MO) level was automatically estimated, or explicitly specified at 50-90.

^b^ Data presented as mean (standard deviation).

^c^ The degrees of freedom is 72.

Table S9. Statistical analysis of network integrity for the PMN/DMN identified using template matching and Hu’s templates.

| Variables | AD | HC | t | p^c^ | Cohen’s d | AUC |
| --- | --- | --- | --- | --- | --- | --- |
| **MO = auto^a^** |  |  |  |  |  |  |
| PMN | 0.27 (0.15)^b^ | 0.45 (0.15) | 4.13 | <0.001 | 1.15 | 0.74 |
| DMN | 0.51 (0.12) | 0.58 (0.093) | 2.25 | 0.027 | 0.65 | 0.67 |
| **MO = 50** |  |  |  |  |  |  |
| PMN | 0.18 (0.093) | 0.29 (0.14) | 3.40 | 0.0011 | 0.94 | 0.71 |
| DMN | 0.48 (0.13) | 0.56 (0.10) | 3.05 | 0.0032 | 0.71 | 0.68 |
| **MO = 60** |  |  |  |  |  |  |
| PMN | 0.20 (0.11) | 0.33 (0.15) | 3.38 | 0.0012 | 0.95 | 0.71 |
| DMN | 0.49 (0.12) | 0.56 (0.11) | 2.60 | 0.011 | 0.61 | 0.66 |
| **MO = 70** |  |  |  |  |  |  |
| PMN | 0.22 (0.11) | 0.38 (0.16) | 4.04 | <0.001 | 1.13 | 0.75 |
| DMN | 0.51 (0.13) | 0.57 (0.099) | 1.91 | 0.060 | 0.53 | 0.64 |
| **MO = 80** |  |  |  |  |  |  |
| PMN | 0.25 (0.14) | 0.42 (0.15) | 4.06 | <0.001 | 1.14 | 0.73 |
| DMN | 0.51 (0.13) | 0.57 (0.11) | 2.34 | 0.022 | 0.52 | 0.67 |
| **MO = 90** |  |  |  |  |  |  |
| PMN | 0.27 (0.14) | 0.43 (0.16) | 3.58 | <0.001 | 1.04 | 0.71 |
| DMN | 0.50 (0.12) | 0.57 (0.10) | 2.52 | 0.014 | 0.57 | 0.68 |

^a^ Model order (MO) level was automatically estimated, or explicitly specified at 50-90.

^b^ Data presented as mean (standard deviation).

^c^ The degrees of freedom is 72.

Table S10. Statistical analysis of network integrity for the PMN/DMN identified using dual regression and Hu’s templates.

| Variables | AD | HC | t | p^c^ | Cohen’s d | AUC |
| --- | --- | --- | --- | --- | --- | --- |
| **MO = auto^a^** |  |  |  |  |  |  |
| PMN | 0.22 (0.055)^b^ | 0.28 (0.054) | 4.68 | <0.001 | 1.17 | 0.78 |
| DMN | 0.18 (0.034) | 0.22 (0.032) | 5.24 | <0.001 | 1.12 | 0.80 |
| **MO = 50** |  |  |  |  |  |  |
| PMN | 0.29 (0.079) | 0.38 (0.064) | 5.31 | <0.001 | 1.31 | 0.80 |
| DMN | 0.29 (0.058) | 0.34 (0.052) | 4.20 | <0.001 | 0.88 | 0.76 |
| **MO = 60** |  |  |  |  |  |  |
| PMN | 0.29 (0.081) | 0.38 (0.059) | 5.30 | <0.001 | 1.28 | 0.79 |
| DMN | 0.28 (0.055) | 0.33 (0.044) | 4.41 | <0.001 | 0.93 | 0.74 |
| **MO = 70** |  |  |  |  |  |  |
| PMN | 0.27 (0.069) | 0.35 (0.062) | 4.88 | <0.001 | 1.18 | 0.77 |
| DMN | 0.29 (0.053) | 0.33 (0.042) | 4.15 | <0.001 | 0.92 | 0.75 |
| **MO = 80** |  |  |  |  |  |  |
| PMN | 0.26 (0.066) | 0.33 (0.061) | 4.98 | <0.001 | 1.20 | 0.79 |
| DMN | 0.23 (0.039) | 0.26 (0.039) | 3.66 | <0.001 | 0.78 | 0.72 |
| **MO = 90** |  |  |  |  |  |  |
| PMN | 0.24 (0.060) | 0.31 (0.057) | 5.03 | <0.001 | 1.22 | 0.79 |
| DMN | 0.23 (0.041) | 0.27 (0.035) | 4.99 | <0.001 | 1.09 | 0.78 |

^a^ Model order (MO) level was automatically estimated, or explicitly specified at 50-90.

^b^ Data presented as mean (standard deviation).

^c^ The degrees of freedom is 72.

_­_

Table S11. Statistical analysis of network integrity for the PMN/DMN/MVN identified using template matching after including nuisance regression in data preprocessing.

| Variables | AD | HC | t | p^c^ | Cohen’s d | AUC |
| --- | --- | --- | --- | --- | --- | --- |
| **MO = auto^a^** |  |  |  |  |  |  |
| PMN | 0.25 (0.14)^b^ | 0.40 (0.15) | 3.58 | <0.001 | 1.04 | 0.71 |
| DMN | 0.42 (0.10) | 0.47 (0.072) | 2.19 | 0.032 | 0.61 | 0.66 |
| MVN | 0.28 (0.12) | 0.33 (0.16) | 1.22 | 0.23 | 0.37 | 0.62 |
| **MO = 50** |  |  |  |  |  |  |
| PMN | 0.30 (0.15) | 0.46 (0.12) | 4.04 | <0.001 | 1.16 | 0.75 |
| DMN | 0.43 (0.12) | 0.47 (0.097) | 1.20 | 0.24 | 0.34 | 0.62 |
| MVN | 0.42 (0.18) | 0.47 (0.22) | 0.32 | 0.75 | 0.28 | 0.60 |
| **MO = 60** |  |  |  |  |  |  |
| PMN | 0.25 (0.15) | 0.42 (0.15) | 4.05 | <0.001 | 1.13 | 0.75 |
| DMN | 0.44 (0.096) | 0.50 (0.084) | 2.72 | 0.0083 | 0.59 | 0.69 |
| MVN | 0.39 (0.18) | 0.45 (0.21) | 0.27 | 0.79 | 0.29 | 0.60 |
| **MO = 70** |  |  |  |  |  |  |
| PMN | 0.27 (0.14) | 0.42 (0.15) | 3.71 | <0.001 | 1.02 | 0.74 |
| DMN | 0.44 (0.099) | 0.49 (0.076) | 2.51 | 0.014 | 0.61 | 0.67 |
| MVN | 0.40 (0.18) | 0.43 (0.22) | 0.15 | 0.88 | 0.17 | 0.60 |
| **MO = 80** |  |  |  |  |  |  |
| PMN | 0.25 (0.14) | 0.41 (0.15) | 4.06 | <0.001 | 1.11 | 0.74 |
| DMN | 0.43 (0.10) | 0.47 (0.080) | 1.4 | 0.16 | 0.37 | 0.63 |
| MVN | 0.29 (0.12) | 0.33 (0.15) | 0.90 | 0.37 | 0.30 | 0.61 |
| **MO = 90** |  |  |  |  |  |  |
| PMN | 0.24 (0.13) | 0.39 (0.15) | 4.14 | <0.001 | 1.08 | 0.74 |
| DMN | 0.37 (0.097) | 0.43 (0.079) | 2.79 | 0.0068 | 0.68 | 0.68 |
| MVN | 0.29 (0.13) | 0.33 (0.15) | 0.76 | 0.45 | 0.30 | 0.61 |

^a^ Model order (MO) level was automatically estimated, or explicitly specified at 50-90.

^b^ Data presented as mean (standard deviation).

^c^ The degrees of freedom is 72.

Table S12. Voxel-wise comparison results for the PMN/DMN/MVN identified using template matching after including nuisance regression in data preprocessing.

| Variables | Total Voxel Size | Significant Voxel Size | Ratio of Significant Voxels (%) | Peak p value (corrected) |
| --- | --- | --- | --- | --- |
| **MO = auto^a^** |  |  |  |  |
| PMN | 2096^b^ | 379 | 18.08 | <0.001 |
| DMN | 3386 | 6 | 0.18 | 0.037 |
| MVN | 2630 | 0 | 0 | 0.35 |
| **MO = 50** |  |  |  |  |
| PMN | 3089 | 163 | 5.28 | <0.001 |
| DMN | 6838 | 0 | 0 | 0.051 |
| MVN | 3340 | 0 | 0 | 0.55 |
| **MO = 60** |  |  |  |  |
| PMN | 2909 | 260 | 8.93 | <0.001 |
| DMN | 6372 | 4 | 0.062 | 0.032 |
| MVN | 3246 | 0 | 0 | 0.21 |
| **MO = 70** |  |  |  |  |
| PMN | 2380 | 224 | 9.41 | 0.0010 |
| DMN | 5485 | 0 | 0 | 0.16 |
| MVN | 3052 | 0 | 0 | 0.12 |
| **MO = 80** |  |  |  |  |
| PMN | 2132 | 296 | 13.88 | <0.001 |
| DMN | 3736 | 0 | 0 | 0.26 |
| MVN | 2571 | 0 | 0 | 0.47 |
| **MO = 90** |  |  |  |  |
| PMN | 1803 | 440 | 24.40 | <0.001 |
| DMN | 2301 | 3 | 0.13 | 0.037 |
| MVN | 2629 | 0 | 0 | 0.65 |

^a^ Model order (MO) level was automatically estimated, or explicitly specified at 50-90.

^b^ Total number of voxels in PMN/DMN networks after thresholded by controlling the local false-discovery rate at p < 0.05.

Table S13. Statistical analysis of network integrity for the PMN/DMN/MVN identified using dual regression after including nuisance regression in data preprocessing.

| Variables | AD | HC | t | p^c^ | Cohen’s d | AUC |
| --- | --- | --- | --- | --- | --- | --- |
| **MO = auto^a^** |  |  |  |  |  |  |
| PMN | 0.29 (0.073)^b^ | 0.36 (0.067) | 4.76 | <0.001 | 1.08 | 0.78 |
| DMN | 0.35 (0.066) | 0.39 (0.045) | 3.19 | 0.0021 | 0.74 | 0.70 |
| MVN | 0.30 (0.093) | 0.33 (0.082) | 1.02 | 0.31 | 0.30 | 0.61 |
| **MO = 50** |  |  |  |  |  |  |
| PMN | 0.35 (0.087) | 0.45 (0.060) | 5.04 | <0.001 | 1.24 | 0.79 |
| DMN | 0.40 (0.080) | 0.46 (0.065) | 3.72 | <0.001 | 0.80 | 0.72 |
| MVN | 0.41 (0.14) | 0.45 (0.16) | 0.62 | 0.54 | 0.26 | 0.61 |
| **MO = 60** |  |  |  |  |  |  |
| PMN | 0.34 (0.083) | 0.42 (0.060) | 4.54 | <0.001 | 1.13 | 0.76 |
| DMN | 0.39 (0.083) | 0.45 (0.066) | 3.38 | 0.0012 | 0.75 | 0.71 |
| MVN | 0.39 (0.12) | 0.42 (0.15) | 0.70 | 0.49 | 0.27 | 0.60 |
| **MO = 70** |  |  |  |  |  |  |
| PMN | 0.31 (0.077) | 0.38 (0.060) | 4.66 | <0.001 | 1.08 | 0.77 |
| DMN | 0.37 (0.081) | 0.42 (0.058) | 2.66 | 0.0096 | 0.67 | 0.67 |
| MVN | 0.36 (0.12) | 0.39 (0.14) | 0.65 | 0.52 | 0.23 | 0.60 |
| **MO = 80** |  |  |  |  |  |  |
| PMN | 0.29 (0.073) | 0.36 (0.065) | 4.79 | <0.001 | 1.09 | 0.79 |
| DMN | 0.34 (0.069) | 0.39 (0.047) | 3.95 | <0.001 | 0.90 | 0.72 |
| MVN | 0.30 (0.093) | 0.33 (0.082) | 0.97 | 0.34 | 0.29 | 0.61 |
| **MO = 90** |  |  |  |  |  |  |
| PMN | 0.27 (0.070) | 0.34 (0.066) | 4.67 | <0.001 | 1.03 | 0.77 |
| DMN | 0.30 (0.055) | 0.33 (0.047) | 3.10 | 0.0027 | 0.64 | 0.69 |
| MVN | 0.29 (0.095) | 0.32 (0.085) | 0.90 | 0.37 | 0.25 | 0.61 |

^a^ Model order (MO) level was automatically estimated, or explicitly specified at 50-90.

^b^ Data presented as mean (standard deviation).

^c^ The degrees of freedom is 72.

Table S14. Voxel-wise comparison results for the PMN/DMN/MVN identified using dual regression after including nuisance regression in data preprocessing.

| Variables | Total Voxel Size | Significant Voxel Size | Ratio of Significant Voxels (%) | Peak p value (corrected) |
| --- | --- | --- | --- | --- |
| **MO = auto^a^** |  |  |  |  |
| PMN | 2096^b^ | 76 | 3.63 | <0.001 |
| DMN | 3386 | 219 | 6.47 | <0.001 |
| MVN | 2630 | 0 | 0 | 0.52 |
| **MO = 50** |  |  |  |  |
| PMN | 3089 | 57 | 1.85 | <0.001 |
| DMN | 6838 | 184 | 2.69 | <0.001 |
| MVN | 3340 | 0 | 0 | 0.79 |
| **MO = 60** |  |  |  |  |
| PMN | 2909 | 53 | 1.82 | <0.001 |
| DMN | 6372 | 273 | 4.28 | <0.001 |
| MVN | 3246 | 0 | 0 | 0.85 |
| **MO = 70** |  |  |  |  |
| PMN | 2380 | 90 | 3.78 | <0.001 |
| DMN | 5485 | 216 | 3.94 | <0.001 |
| MVN | 3052 | 0 | 0 | 0.70 |
| **MO = 80** |  |  |  |  |
| PMN | 2132 | 55 | 2.58 | 0.0024 |
| DMN | 3736 | 161 | 4.31 | <0.001 |
| MVN | 2571 | 0 | 0 | 0.58 |
| **MO = 90** |  |  |  |  |
| PMN | 1803 | 78 | 4.33 | 0.0010 |
| DMN | 2301 | 192 | 8.34 | <0.001 |
| MVN | 2629 | 0 | 0 | 0.53 |

^a^ Model order (MO) level was automatically estimated, or explicitly specified at 50-90.

^b^ Total number of voxels in PMN/DMN networks after thresholded by controlling the local false-discovery rate at p < 0.05

Table S15. Comparisons between potential sub-groups of AD in demographics, brain volumetric and neuropsychological assessments.

| Variables | AD (N=7) | AD (N=29) | t | df^d^ | p |
| --- | --- | --- | --- | --- | --- |
| **Demographics** |  |  |  |  |  |
| age in years | 67.00 (7.66) ^a^ | 68.21 (8.14) | 0.22 | 33 | 0.83 |
| education years | 10.43(3.69) | 9.10(4.73) | 0.72 | 33 | 0.48 |
| **Brain Volumetric** | |  |  |  |  |
| intracranial volume (cm^3^) | 1361.02 (120.65) | 1293.32 (102.94) | 1.21 | 33 | 0.24 |
| gray matter (%) | 50.40 (2.19) | 47.34 (2.94) | 3.14 | 33 | 0.0036 |
| white matter (%) | 30.56 (2.29) | 29.19 (3.10) | 0.65 | 33 | 0.52 |
| CSF (%) | 19.04 (2.89) | 23.47 (4.15) | 2.61 | 33 | 0.013 |
| hippocampi (%) | 0.48 (0.094) | 0.44 (0.079) | 1.04 | 33 | 0.31 |
| **Neuropsychological Assessments** | |  |  |  |  |
| MMSE | 19.43 (4.12) | 16.28 (6.35) | 1.70 | 33 | 0.099 |
| MoCA^b^ | 15.57 (5.16) | 11.96 (5.29) | 2.03 | 31 | 0.051 |
| AVLT-IR^c^ | 3.90 (2.00) | 3.42 (1.65) | 1.12 | 32 | 0.27 |
| AVLT-DR^c^ | 1.71 (2.21) | 0.79 (1.52) | 1.08 | 32 | 0.29 |

^a^ Data presented as mean (standard deviation).

^b^ Missing data of two subjects for MoCA.

^c^ AVLT-IR and AVRT-DR represent immediate recall and delayed recall of AVLT. Missing data of one subject for AVLT.

^d^ df stands for degrees of freedom.
